# Antimicrobial and antioxidant activities of water‐soluble extracts of Camis cheeses produced by different traditional methods

**DOI:** 10.1002/fsn3.4305

**Published:** 2024-07-02

**Authors:** Irem Uzunsoy

**Affiliations:** ^1^ Çaycuma Vocational School of Food and Agriculture Zonguldak Bülent Ecevit University Zonguldak Turkey

**Keywords:** antimicrobial, antioxidant, Buffalo, Camis cheese, ripening time, water‐soluble extract

## Abstract

Traditional Camis cheese may be an interesting dairy product with potential bioactivities and postconsumption health effects. The aim of this study was to evaluate the antimicrobial and antioxidant activity of Camis cheeses produced by two different traditional methods, differing in heat processing of the milk and boiling the curd, and to reveal the changes that occur depending on 90 days of ripening time. For this purpose, physicochemical and sensory analyses were performed, and antimicrobial and antioxidant activity analyses of the water‐soluble extracts (WSEs) of Camis cheeses (B1 and B2) were conducted. WSEs of both cheeses (800 μL mL^−1^) had remarkable antimicrobial effects on *Escherichia coli* (*E. coli*) and *Staphylococcus aureus* (*Staph. aureus*), with a higher inhibitory effect on *E*. *coli*. The Trolox equivalent antioxidant capacity (TEAC) values (0.12–2.24 μM Trolox) and % inhibition rates as 2,2‐diphenyl‐1‐picrylhydrazil (DPPH) radical scavenging activity (8.54–26.34%) of the cheeses indicate limited antioxidant activity. The curd boiling process was found to have a favorable effect on the antimicrobial and antioxidant activity of the Camis cheeses. However, the panelists' perceptions of flavor, texture, and total acceptability did not align with this positive outcome. The results of this study reveal that the consumption of traditional Camis cheese and its extracts or the use of purified bioactive peptides in functional food products may constitute an important alternative in terms of eliminating the negative effects encountered with the use of synthetic antimicrobial and antioxidant components to provide the desired health effect from natural sources.

## Graphical abstract

The antimicrobial and antioxidant activities of Camis cheeses produced by two different traditional methods, differing in heat processing of the milk and boiling the curd, were evaluated during 90 days of ripening time. Water‐soluble extracts possessed limited antioxidant activity but strong antimicrobial activity against *Escherichia coli* (ATCC® 25922™) and *Staphylococcus aureus* (ATCC® 23235™) at a concentration of 800 μL mL^−1^. High heat treatment and a lack of starter culture affected the bioactivity of cheeses.

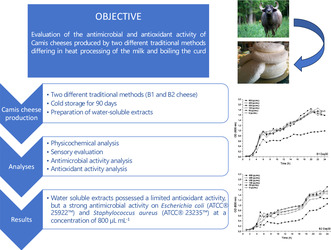

## 1 INTRODUCTION

**FIGURE 1:**
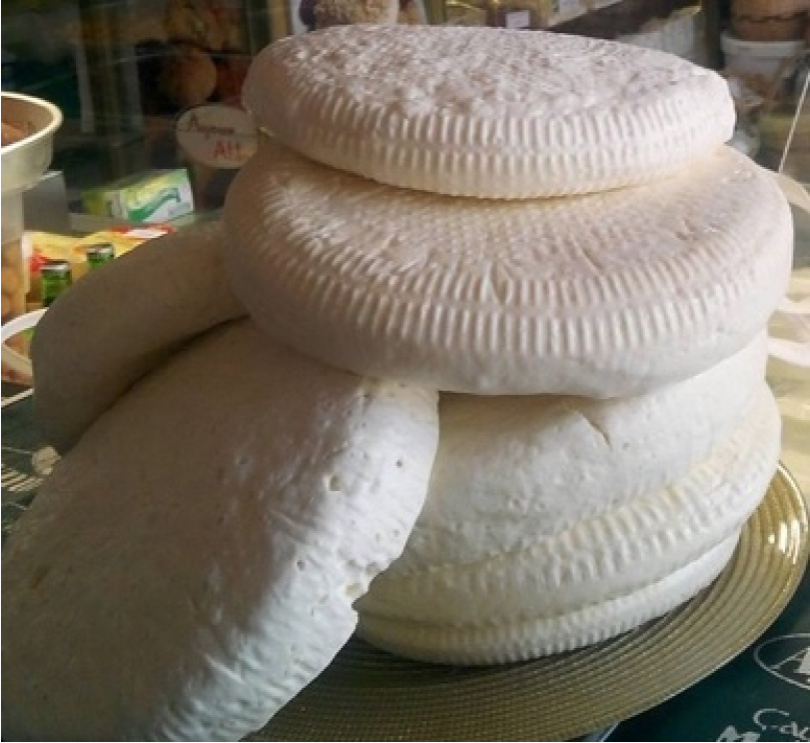
Camis cheese.

Recently, consumers have preferred traditional products that have postconsumption health effects in addition to the expected taste and nutritional value. In this context, it is very important to reveal the positive effects of these products on health. Camis (also known as Camiz) cheese is a traditional, aromatic, and delicious buffalo cheese in the Black Sea Region, with a semi‐hard/hard texture (Figure 1). For the cheesemaking process, first unpasteurized buffalo milk is heated at 50–75°C for 5 min for the inactivation of endogenous milk enzymes and undesired microflora. After cooling to 30–35°C, rennet is added and left for curdling. Then the curd is cut, filtered through cheesecloth, and placed into a perforated basket for molding or boiled in heated whey and filtered again. It is marketed fresh as well as brined for ripening period.

Due to their negative health effects on humans, the use of antimicrobial additives is limited, and recently, fermentation and/or the addition of natural antimicrobial components have been prioritized to eliminate these negative effects. Naturally occurring peptides and proteins may exhibit activity comparable to antibiotics and represent a potential natural alternative against several bacteria like *Staphylococcus*, *Escherichia*, and *Listeria* (Ji et al., 2024).

Free radicals and reactive oxygen species (ROS) formed as a result of oxidation reactions lead to the development of various degenerative disorders such as heart attack, atherosclerosis, and cancer (Abd El‐Fattah et al., 2020). Besides, ROS cause undesirable effects on the sensory properties of foods, thereby reducing their shelf life, nutritional quality, and food safety. Therefore, controlling oxidative stress is very important in slowing the progression of diseases, preventing complications, and maintaining the quality of foods. In addition to natural and synthetic antioxidants, peptides obtained from different sources also draw attention as natural antioxidant components.

Cheese is a rich source of bioactive peptides released during production and ripening processes (Santiago‐López et al., 2018). The formation of bioactive peptides in cheese is influenced by milk type, the use of starter culture, heat treatment, and ripening conditions. Different types of cheeses like Minas (Fialho et al., 2018) and fresh buffalo cheese (Silva et al., 2019) show extensive inhibition against Gram‐positive and Gram‐negative bacteria, molds, and yeasts. Also, strong antioxidant activity was determined in Cheddar (Huma et al., 2018) and Kalari cheese (Mushtaq et al., 2016).

Although there are many studies examining the bioactivity of different types of cheese from different milk types, studies on buffalo cheeses are very limited. The results of studies on the antimicrobial activity of Mozzarella (Théolier et al., 2014) and the antioxidant activity of Kalari (Mushtaq et al., 2016), Cheddar (Huma et al., 2018), and fresh buffalo cheeses (Ribas et al., 2019) show significant bioactivity. Hence, this study aimed to evaluate the bioactivity of Camis cheeses produced by two different traditional methods in terms of antimicrobial and antioxidant activities and to reveal the changes that occur depending on the ripening time.

## 2 MATERIALS AND METHODS

### 2.1 Materials

Camis cheesemaking processes were carried out on two different local dairy farms. Microbial rennet (Chr. Hansen A/S, Hoersholm, Denmark) was used for curdling, and brine salt for salting. Bacterial strains *E. coli* (ATCC® 25922™) and *Staph. aureus* (ATCC® 23235™) used in this study were obtained from Hemakim Corporation (Istanbul, Turkey). All chemicals were provided by Merck KGaA (Darmstadt, Germany) unless stated otherwise.

### 2.2 Cheesemaking and sampling

The Camis cheeses (B1 and B2) were produced by two different traditional methods, mainly differing in heat processing of the milk and boiling the curd, since the degree of heat treatment affects the ripening period by altering the natural microflora and endogenous enzyme activities, hence the amount and diversity of bioactive peptides. For cheese B1, previous heat treatment of unpasteurized buffalo milk (50–55°C, 5 min) and cooling (30–35°C) were done before rennet addition (1.0–1.5 mL L^−1^ milk). After curdling and cutting, filtered curd was boiled in whey (90–95°C) for 5 min and taken into a perforated basket for whey drainage. For cheese B2, unpasteurized buffalo milk was heated at 70–75°C for 5 min, followed by a cooling step (30–35°C) and rennet addition (1.0–1.5 mL L^−1^ milk). After the curdling was completed, it was cut and left for whey drainage. Then the whey was filtered through cheesecloth and taken into a perforated basket with a fivefold weight on it. Cheesemaking was performed in triplicate.

Fresh B1 and B2 cheeses were submerged in 12% brine and kept at 4–6°C for 15 days before vacuum packaging. The cold storage of the cheeses was continued for 90 days. WSEs were prepared and stored at −20°C until the bioactivity analyses were conducted. Sampling and all analyses were performed in triplicate on the 1, 30, 60, and 90th days of ripening.

### 2.3 Physiochemical analysis

The titratable acidity expressed as lactic acid (l.a, %), pH, dry matter, ash, fat, salt, and total nitrogen (TN) were carried out on the buffalo milk and Camis cheese samples (AOAC, 2012). Protein content was found by multiplying TN content by the coefficient of 6.38.

### 2.4 Sensory analysis

Sensory evaluations were made utilizing a balanced 9‐point scale ranging from 1 (disliked) to 9 (the most liked), with 30 panelists of both genders working and studying at Zonguldak Bülent Ecevit University (Zonguldak, Turkey) who consume cheese regularly. Before the test, informed consent was obtained from each panelist before they participated in the study, and then oral instructions were given on how to conduct the test. Panelists put forward their preferences in order of flavor, texture, and total acceptability. Odorless and tasteless crackers and water were also given to cleanse their palates between samples.

### 2.5 Preparation of WSEs

For the preparation of WSEs, the method developed by Théolier et al. (2014) was slightly modified. At first, 100 g of blended cheese sample was homogenized with 300 mL of deionized water at 1000 rpm for 10 min, kept in a water bath at 40°C for 1 h, and then centrifuged at 4000 x *g* at 4°C for 30 min. After centrifugation, the upper fat layer was separated, the supernatant obtained was filtered through Whatman No. 2, and its pH was adjusted to 4.6 with 1 N HCl. Then the precipitate was separated by centrifugation (at 4000 x *g*, 30 min at 4°C), and the filtrate was filtered through a 0.22 μm pore diameter microfilter and stored at −20°C until use.

### 2.6 Determination of the antimicrobial activity

The antimicrobial activity of WSEs was evaluated for *E*. coli (ATCC® 25922™) and *Staph. aureus* (ATCC® 23235™) bacteria.

#### 2.6.1 The disc diffusion method

With slight modifications in the zone of inhibition method (CLSI, 2012), each bacterium was activated and plated on Petri dishes containing Mueller–Hinton agar. Wells were created on the agar by using the reverse of a sterile Drigalski loop and filled with 100 μL of WSE. Positive (streptomycin) and negative (pure water) control samples were also prepared under the same conditions. All samples were incubated at 37°C for 24 h, and the inhibition zone diameter (mm) formed after incubation was measured.

#### 2.6.2 The tube dilution method

For the determination of the antimicrobial activity of WSEs, the tube dilution method was also used with some modifications (Mushtaq et al., 2021). Each WSE was prepared at concentrations ranging from 50 to 800 μL mL^−1^ in Tryptic Soy Broth (TSB), inoculated with 1% (v/v) of each bacterium, incubated at 37°C for 24 h, and optical density at 600 nm was measured every hour with a UV–Vis spectrophotometer (PG Instruments Ltd., Leicestershire, UK). Thus, 24‐hour growth was observed, and antimicrobial activity was evaluated by comparing it with the control sample, the bacteria inoculated and incubated in TSB at the same conditions without the WSEs.

### 2.7 Determination of the antioxidant activity

#### 2.7.1 ABTS method

The antioxidant activity is determined as the percent inhibition of absorbance at 734 nm, with the ABTS method developed by Re et al. (1999). ABTS^•+^ radical was prepared by taking 5 mL of 7 mM ABTS stock solution and mixing it with 88 μL of 140 mM K_2_ S_2_O_8_ (final concentration 2.45 mM) and kept in an amber‐colored bottle for 12–16 h at room temperature and dark before use. The prepared solution was diluted with 0.1 M phosphate buffer solution (PBS, pH 7.4) to 0.7 ± 0.02 absorbance at 734 nm and equilibrated at 30°C. 2 mL of the diluted ABTS^•+^ solution was mixed with 20, 30, and 40 μL of WSE or different concentrations of Trolox standard (in PBS) as a positive control (12.5–400 μM). Immediately after mixing, the sample was placed in the UV–Vis spectrophotometer at 30°C, and absorbance was measured at 734 nm when placed and per minute for 6 min. The % inhibition values of the sample and Trolox were determined, and the TEAC (μM Trolox) values of the samples were obtained by dividing the slope of the % inhibition rate curve depending on the sample concentration by the slope of the % inhibition rate curve depending on the Trolox concentration, as follows (1 and 2).

(1) $$\text{Inhibition rate}\;\left(\%\right)=\frac{A_{C}-A_{\mathrm{WSE}}}{A_{C}}\times 100$$

where *A*_C_ is the absorbance of the Trolox standard and *A*_WSE_ is the absorbance of WSE.

(2) $$\text{TEAC}\;\left(\frac{\mathrm{mM}\;\text{Trolox}}{g\;\text{sample}}\right)=\left(\frac{a}{b}\right)\times \mathrm{DF}$$

where *a* is the slope of inhibition curve of sample, *b* is the slope of Trolox standard curve, and DF is the dilution factor.

#### 2.7.2 DPPH method

For the determination of antioxidant activity with DPPH, the method used by Erkaya (2014) was modified. For this purpose, 1.5 mL of WSE was mixed with 1.5 mL of freshly prepared 60 μM DPPH (in 95% methanol) and kept in the dark for 45 min at room temperature. Then, absorbance was measured at 517 nm with UV–Vis spectrophotometer, and the radical scavenging activity of the WSEs was calculated as follows (3):

(3) $$\text{DPPH radical scavenging activity}\;\left(\%\right)=\left(1-\frac{A_{\mathrm{WSE}}}{A_{B}}\right)\times 100$$

where *A*_B_ is the absorbance of the blind sample (deionized water) and *A*_WSE_ is the absorbance of WSE.

#### 2.7.3 Total phenolic compound analysis

The method used by Chávez‐Servín et al. (2018) was modified for the determination of total phenolic compounds. One milliliter of WSE was mixed with 1 mL of ethanol (95%, v/v), 5 mL of distilled water, and 0.5 mL of 50% (v/v) Folin–Ciocalteu reagent (in deionized water), respectively. After 5 min, 1 mL of Na_2_CO_3_ (5%, w/v) was added to the mixture and held for 60 min. Then, absorbance was measured at 725 nm with a UV–Vis spectrophotometer, and the TPC of WSEs was calculated on a standard curve constructed using gallic acid (95% (w/v), ethanol, 6.25–100 μg mL^−1^) and was expressed as milligrams of gallic acid equivalents per mL (mg of GAE mL^−1^).

### 2.8 Statistical analysis

Statistical analyses were performed by SPSS Statistics (v.22.0). The differences between the ripening days of the same samples were compared by the one‐way analysis of variance (ANOVA) (*p* > .05) followed by Tukey's/Games‐Howell post hoc tests, and the differences between the cheese samples at the same ripening days were analyzed by an independent samples t‐test. Data were expressed as mean values ± standard deviation (SD) when indicated.

## 3 RESULTS AND DISCUSSION

### 3.1 Physicochemical properties of buffalo milk and Camis cheese samples

Tables 1 and 2 show the physicochemical analysis results of buffalo milk and Camis cheese samples, respectively. The pH values of B1 cheese on the 30th and 90th days were found to be statistically different (*p* < .05). The titratable acidity (l.a., %), ash, salt, and salt content on a dry matter basis, dry matter, milk fat, and milk fat on a dry matter basis, TN, and protein contents of B1 cheese were similar between ripening days (*p* > .05). There was no statistically significant difference in pH values, titratable acidity (l.a., %), ash, dry matter, milk fat, and milk fat on a dry matter basis, TN, and protein contents of B2 cheese during ripening (*p* > .05). The salt content and salt content on dry matter basis of B2 cheese varied considerably between the 1st and 30th days of ripening due to brining as expected.

**TABLE 1:** Physicochemical properties of buffalo milk samples.

Physicochemical properties	BM1	BM2
pH	6.59 ± 0.03^B^	6.71 ± 0.01^A^
Titratable acidity		
l.a. (%)	0.26 ± 0.06^A^	0.19 ± 0.00^A^
SH	12.94 ± 2.83^A^	10.54 ± 0.12^A^
Milk fat (%)	7.12 ± 0.56^A^	8.01 ± 0.63^A^
Total nitrogen (%)	0.69 ± 0.07^A^	0.55 ± 0.14^A^
Protein (%)	4.37 ± 0.48^A^	3.49 ± 0.88^A^

*Note*: The upper case letters in the same line (^A,B^) indicate that the differences between BM1 and BM2 milk are significant (*p* < .05).

Abbreviations: BM1, buffalo milk used in B1 cheesemaking; BM2, buffalo milk used in B2 cheesemaking; l.a., lactic acid; SH, Soxhlet‐Henkel.

**TABLE 2:** Physicochemical properties of Camis cheese samples.

Physicochemical properties	Ripening period (days)			
	1	30	60	90
pH				
B1	6.84 ± 0.15^bA^	7.04 ± 0.01^aA^	6.65 ± 0.26^bA^	6.67 ± 0.01^bA^
B2	6.19 ± 0.22^aB^	6.35 ± 0.08^aB^	6.39 ± 0.16^aA^	6.35 ± 0.17^aB^
Titratable acidity (l.a., %)				
B1	0.27 ± 0.15^aA^	0.22 ± 0.02^aA^	0.22 ± 0.08^aA^	0.09 ± 0.05^aA^
B2	0.22 ± 0.23^aA^	0.09 ± 0.04^aB^	0.12 ± 0.10^aA^	0.15 ± 0.16^aA^
Ash (%)				
B1	5.14 ± 2.12^aA^	5.49 ± 0.86^aA^	6.38 ± 1.13^aA^	8.25 ± 1.80^aA^
B2	4.09 ± 1.60^aA^	4.44 ± 1.39^aA^	4.82 ± 0.99^aA^	6.08 ± 1.39^aA^
Salt (%)				
B1	2.31 ± 1.91^aA^	4.76 ± 0.31^aA^	4.56 ± 0.81^aA^	5.03 ± 0.94^aA^
B2	1.49 ± 0.78^bA^	4.27 ± 1.02^aA^	3.25 ± 0.61^aA^	3.46 ± 0.82^aA^
Salt on dry matter basis (%)				
B1	4.91 ± 4.08^aA^	9.82 ± 0.74^aA^	9.33 ± 1.29^aA^	10.07 ± 1.40^aA^
B2	2.19 ± 1.14^bA^	7.00 ± 1.98^aA^	5.25 ± 1.36^aB^	5.35 ± 1.89^aB^
Dry matter (%)				
B1	47.09 ± 0.30^aA^	48.49 ± 1.78^aA^	48.65 ± 1.89^aA^	49.75 ± 3.15^aA^
B2	67.48 ± 2.73^aB^	61.84 ± 5.25^aB^	62.65 ± 4.21^aB^	66.50 ± 9.97^aB^
Milk fat (%)				
B1	22.92 ± 3.74^aA^	22.50 ± 1.80^aA^	24.75 ± 3.75^aA^	28.33 ± 3.33^aA^
B2	39.17 ± 1.26^aB^	36.50 ± 1.32^aB^	34.92 ± 2.13^aB^	37.92 ± 2.77^aB^
Milk fat on dry matter basis (%)				
B1	48.70 ± 8.23^aA^	46.35 ± 1.99^aA^	50.80 ± 6.52^aA^	56.83 ± 3.46^aA^
B2	58.05 ± 0.90^aA^	59.21 ± 3.50^aB^	55.92 ± 2.08^aA^	57.47 ± 4.56^aA^
Total nitrogen (%)				
B1	1.49 ± 0.12^aA^	1.91 ± 0.45^aA^	2.07 ± 0.60^aA^	2.29 ± 0.67^aA^
B2	2.54 ± 0.69^aA^	2.59 ± 0.70^aA^	2.20 ± 0.54^aA^	2.15 ± 0.52^aA^
Protein (%)				
B1	9.48 ± 0.79^aA^	12.18 ± 2.88^aA^	13.20 ± 3.85^aA^	14.63 ± 4.29^aA^
B2	15.85 ± 4.20^aA^	16.55 ± 4.47^aA^	14.02 ± 3.43^aA^	13.70 ± 3.34^aA^

*Note*: The lower case letters in the same line (a, b) indicate that the differences between the ripening days of the same cheese sample are significant (*p* < .05). The upper case letters in the same column (A, B) indicate that the differences between B1 and B2 cheeses on the same ripening day are significant (*p* < .05).

Abbreviations: l.a., lactic acid; B1 and B2, Camis cheeses produced with two different traditional methods.

When B1 and B2 cheeses were compared, there were no statistically significant differences between the ash, salt, TN, and protein contents between B1 and B2 cheeses on all ripening days (*p* > .05). The pH values of the two cheeses except on day 60, titratable acidity on day 30, salt on a dry matter basis on days 60 and 90, dry matter and milk fat on all ripening days, and milk fat on a dry matter basis on day 30 were significantly different (*p* < .05). The acidity of B2 cheese decreased on day 30 and then increased during ripening, which was also observed in Kashar cheese produced from buffalo milk during 90 days of ripening (Okumus, 2019). The dry matter contents of B2 cheese were rather higher than those of B1 cheese due to pressing during whey filtration at the last stage of production. The protein content increase between days 1 and 30 is thought to be due to the moisture loss of the samples due to the osmotic effect caused by the salt concentration of the brine during the 15‐day brine holding. The protein values of the 1st and 30th‐day samples of B1 cheese were found to be lower than cheeses produced from buffalo milk in the literature (Okumus, 2019).

### 3.2 Sensory evaluation

The changes in flavor, texture, and total acceptability scores of the cheeses through the 90 days of ripening are given in Table 3. There was no statistically significant difference in flavor, texture, and total acceptability of B1 cheese between ripening days (*p* > .05). Panelists' perceptions of flavor, texture, and total acceptability decreased slightly in the 30th‐day sample and then showed an increasing trend for B1 cheese. The panelists interpreted that the salty taste predominant in 30th‐day samples, in line with the increase in the salt amount due to brining, caused the flavor and total acceptability scores to decrease. Despite this negativity, the flavor and texture scores increased during ripening, and the total acceptability of the samples improved due to ripening.

**TABLE 3:** The sensory scores of Camis cheese samples during 90 days of ripening.

Sensory properties	Ripening period (days)			
	1	30	60	90
Flavor				
B1	4.97 ± 2.61^A^	4.77 ± 1.89^A^	5.33 ± 2.16^A^	5.13 ± 2.56^A^
B2	5.27 ± 2.89^A^	5.70 ± 2.51^A^	4.90 ± 2.78^A^	4.70 ± 2.90^A^
Texture				
B1	4.93 ± 2.30^A^	4.53 ± 1.83^B^	5.57 ± 2.33^A^	5.70 ± 2.49^A^
B2	5.27 ± 2.63^A^	6.03 ± 2.22^A^	5.10 ± 2.73^A^	5.43 ± 2.96^A^
Total acceptability				
B1	4.90 ± 2.38^A^	4.73 ± 1.98^A^	4.83 ± 2.41^A^	4.80 ± 2.64^A^
B2	5.00 ± 2.85^A^	5.63 ± 2.57^A^	4.97 ± 2.82^A^	4.73 ± 3.03^A^

*Note*: The upper case letters in the same column (^A,B^) indicate that the differences between B1 and B2 cheeses on the same ripening day are significant (*p* < .05).

Abbreviation: B1 and B2, Camis cheeses produced with two different traditional methods.

In contrast, the flavor, texture, and total acceptability scores of B2 cheese increased in 30th‐day sample but showed a descending trend until the end of ripening. Similarly, there was no statistically significant difference between the sensory evaluation results of B1 and B2 in all ripening days (*p* > .05), except for the textural evaluation scores of the 30th day samples (*p* < .05). The panelists stated that B2 cheese was preferred in terms of hardness and springiness due to the higher dry matter content of B2 cheese (Table 2). Up to day 30, the flavor and texture scores of B2 cheese were slightly higher than those of B1, and then the scores of B1 cheese increased during ripening. This change was also seen in total acceptability scores after day 60. From all the evaluation results, it is understood that the cheesemaking process of B1 cheese with curd boiling will be more advantageous in terms of consumer preferences for Camis cheeses ripened for at least two months.

### 3.3 Antimicrobial activity

The antimicrobial activity results obtained by the disc diffusion method are given in Table 4. It was seen that only the WSEs of the B1 cheese showed antimicrobial activity against *E. coli* (ATCC® 25922™). While the inhibition zone diameter was 4.66 mm on the 1st day, it decreased to 4.30 mm on the 30th day but almost doubled to 8.50 mm on the 60th day. A small zone of inhibition (1.45 mm) was observed on day 90. While no inhibition zone was formed in pure water, a 46‐mm zone was observed in the presence of streptomycin.

**TABLE 4:** The antimicrobial activity results of Camis cheese samples during 90 days of ripening (disc diffusion method).

Bacteria	Ripening day	Inhibition zone diameter (mm)	
		B1	B2
*Escherichia coli* (ATCC 25922)	1	4.66 ± 0.06^b^	0
	30	4.30 ± 0.27^b^	0
	60	8.50 ± 0.30^a^	0
	90	1.45 ± 0.04^c^	0
*Staphylococcus aureus* (ATCC 23235)	1	0	0
	30	0	0
	60	0	0
	90	0	0

*Note*: The lower case letters in the same column (^a,b^) indicate that the differences between the ripening days of the same cheese sample are significant (*p* < .05). While no inhibition zone was formed in pure water, a 46‐mm zone was observed in the presence of streptomycin.

Abbreviation: B1 and B2, Camis cheeses produced with two different traditional methods.

The changes observed during ripening can be explained by the change in the amount and type of antimicrobial peptides released by proteolysis during cheese production and ripening. The partial decrease in the inhibition zone observed at day 30 and the noticeable decrease at day 90 are related to the further hydrolysis of some of the antimicrobial peptides during ripening (Albenzio et al., 2017). New antimicrobial peptides released by secondary proteolysis during the ripening period increase the antimicrobial activity and the diameter of the inhibition zone. The decrease in the inhibition zone at day 90 is an indication that antimicrobial peptides are mostly degraded after day 60. It was observed that the 7‐day‐old Kalari WSEs formed an inhibition zone of 5.11 mm on *E. coli* (ATCC® 25922™) (Mushtaq et al., 2021). These results are similar to the inhibition zones of B1 cheese WSEs on *E. coli* (ATCC® 25922™), especially those formed by the 1st and 30th ripening day samples. No antimicrobial activity on *E. coli* (ATCC® 25922™) was detected in B2 cheese samples, as on *E*. *coli* (ATCC® 11303™) in Orgu cheese (Canozer & Kose, 2022) and *E. coli* (ATCC® 25922™) in fresh buffalo cheese (Silva et al., 2019). The antimicrobial activity of WSEs from all ripening days of both cheese samples on *Staph. aureus* (ATCC® 23235™) could not be determined by the disc diffusion method. No inhibition zones were formed after incubation with the addition of WSEs to the *Staph. aureus*‐developed petri dishes. In similar studies, extracts of Mozzarella cheese (Yerlikaya et al., 2021) and fresh buffalo cheese (Silva et al., 2019) did not contain fractions showing antimicrobial activity and did not form inhibition zones against *Staph*. *aureus* (ATCC® 12600™) and *Staph. aureus* (ATCC® 6538™), respectively. However, there are also studies indicating the antimicrobial effect of WSEs of Kalari cheese on *Staph. aureus* (ATCC® 23235™) (5.16 mm, Mushtaq et al., 2021). The formation of the inhibition zone varies depending on the presence of antimicrobial peptides as well as the inhibitory effect of these peptide fractions on the selected indicator microorganism.

**FIGURE 2:**
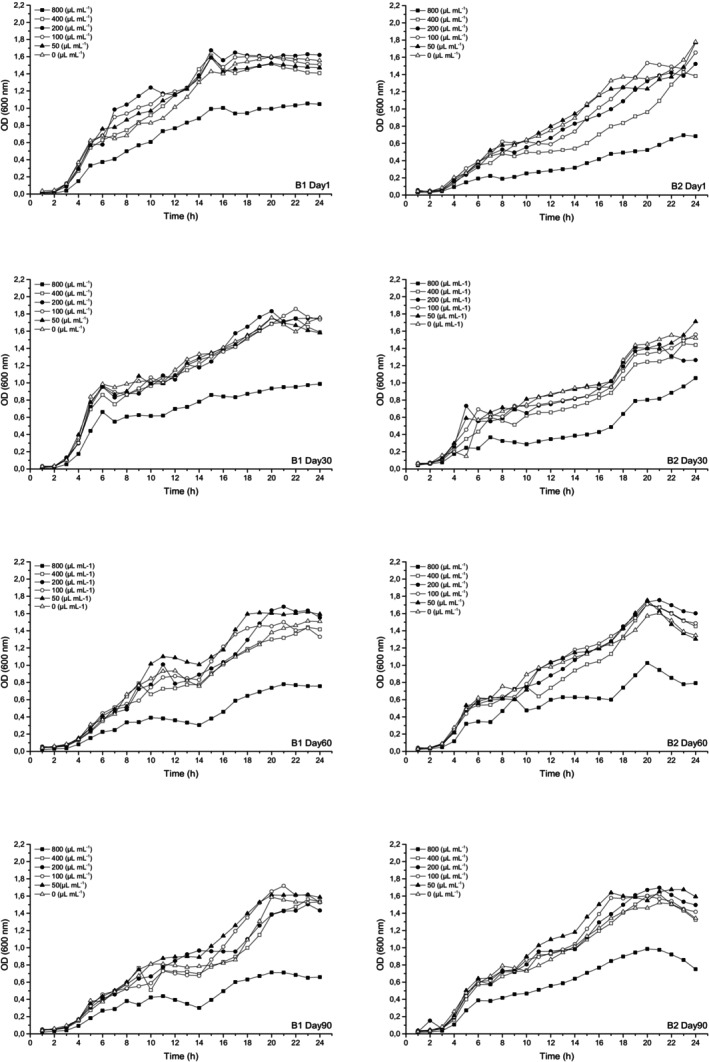
Growth curve of *E. coli* (ATCC 25922) in TSB with different concentrations (0–800 μL mL^−1^) of B1 and B2 WSEs during ripening.

**FIGURE 3:**
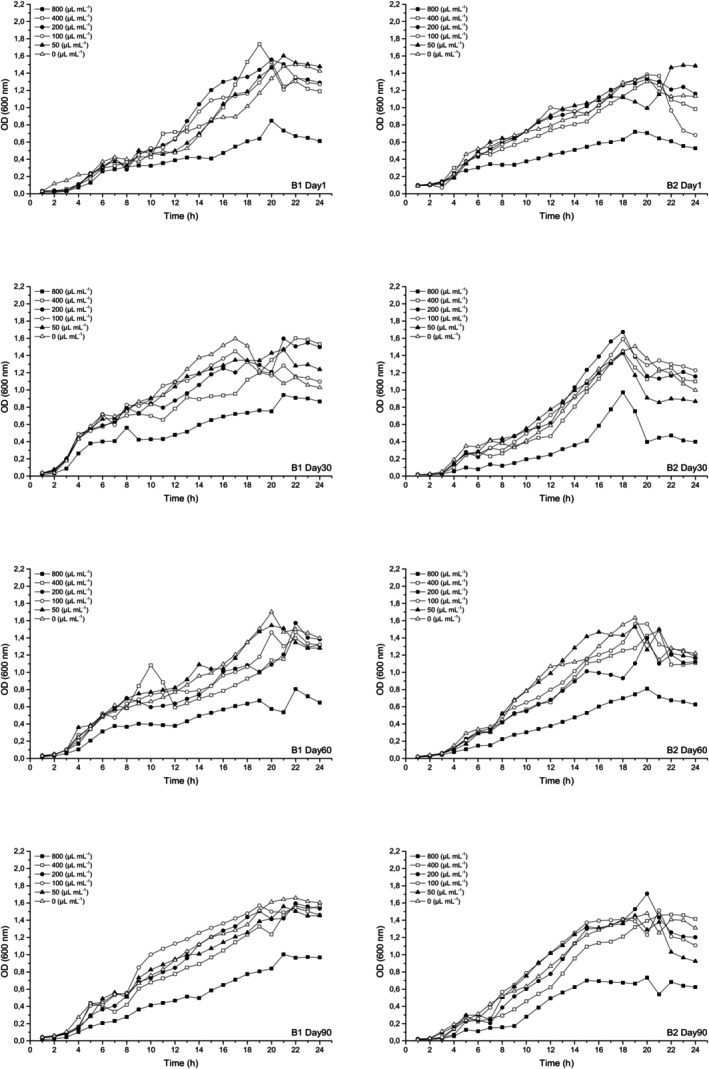
Growth curve of *Staph. aureus* (ATCC 23235) in TSB with different concentrations (0–800 μL mL^−1^) of B1 and B2 WSEs during ripening.

When the 24‐hour bacterial growth curves in the presence of WSEs were analyzed (Figures 2 and 3), it was observed that the bacterial growth during the ripening period in all cheese samples at the highest WSE concentration (800 μL mL^−1^) was noticeably lower than in the control sample without WSE. Bacterial inhibition was similar in B1 and B2 cheeses, except that growth was lower in B2 cheese on days 1 and 30 of ripening, and antimicrobial activity was maintained for both bacteria during ripening. B2 cheese showed higher activity than B1 cheese until day 60. For *Staph. aureus* (ATCC® 23235™) a decrease in inhibition was evident in B1 and B2 during ripening. New antimicrobial peptides are formed by proteolysis, and some of these peptides are degraded during ripening, resulting in a decrease in antimicrobial activity (Albenzio et al., 2017). At concentrations below 800 μL mL^−1^ (50–400 μL mL^−1^) of both cheese samples, inhibition increased at certain times during 24‐h development, and bacterial growth was reduced compared to the control sample. In general, the inhibitory activity of WSEs can be more clearly observed in the stationery and death phases during the growth of the target bacteria. It is thought that bacterial growth was somewhat supported due to the composition of WSEs at 50–400 μL mL^−1^ concentrations in some ripening days. Similarly, some studies have shown the stimulatory effect of peptides in cheese WSEs on *E. coli* BC402 (Erkaya, 2014). However, the stimulation/inhibition effect changes during ripening. It can also be observed that, in general, the concentrations of WSE studied have higher antimicrobial effects on *Staph. aureus* (ATCC® 23235™) than on *E. coli* (ATCC® 25922™).

The type and number of antimicrobial peptides released in cheeses vary depending on the type of milk used in production, the type of cheese, and the ripening process. Peptide extracts of Cheddar cheese showed antimicrobial activity against *B. cereus*, *E. coli*, and *Staph. aureus* (Nguyen Thi et al., 2014), while WSE of Canastra cheese samples was effective against *E. coli* (Fialho et al., 2018). Moreover, Gouda and Mozzarela WSEs showed antimicrobial activity against *E. coli* MC4100 and *E. coli* O157:H7 (Théolier et al., 2014). No antimicrobial effect was detected on *Salmonella typhimurium* (ATCC® 14028™) and *E*. *coli* (ATCC® 25922™) in WSEs of cow cheeses (Turan & Durak, 2022). During the ripening of cheeses, changes in antimicrobial activity can be observed depending on the type and number of peptides on different ripening days. In moderately ripened Cheddar cheeses, *Listeria ivanovii* inhibition activity was high but decreased as ripening time progressed (Théolier et al., 2014).

### 3.4 Antioxidant activity

The antioxidant activity results of the Camis cheese samples are given in Table 5. It was hard to find comparable data in the literature on the antioxidant activity of buffalo cheeses made with the temperature–time combinations close to this study, such as curd boiling in whey at high temperatures. For the ABTS assay, all cheese samples had low levels of antioxidant activity (1.39–4.69 μM Trolox). The TEAC values obtained on all ripening days for both B1 and B2 cheeses were lower than those of Orgu cheeses produced by industrial and traditional methods (2.70 ± 0.73, 3.19 ± 0.70 mM Trolox, respectively; Canozer & Kose, 2022) and white brined goat cheese (35–55 mM Trolox, Barać et al., 2016). The lack of starter culture addition, which would contribute to the ripening process by proteolysis for the release of antioxidative peptides, might explain the low TEAC values (Mushtaq et al., 2019).

**TABLE 5:** The antioxidant capacity of Camis cheese samples during 90 days of ripening.

Analyses	Ripening day	B1	B2
ABTS assay			
Inhibition (%)	1	2.35 ± 1.02^aA^	2.58 ± 0.85^aA^
	30	2.63 ± 2.11^aA^	3.34 ± 0.74^aA^
	60	2.32 ± 0.48^aA^	2.81 ± 0.42^aA^
	90	3.22 ± 1.19^aA^	2.78 ± 0.40^aA^
TEAC value (μM Trolox)	1	1.97 ± 0.44^aA^	1.39 ± 0.63^aA^
	30	2.06 ± 0.46^aA^	3.46 ± 0.97^aA^
	60	1.88 ± 0.54^aA^	1.44 ± 0.46^aA^
	90	4.69 ± 0.84^aA^	1.85 ± 0.03^aA^
DPPH assay			
Inhibition (%)	1	11.58 ± 1.09^bB^	15.06 ± 0.62^bA^
	30	23.35 ± 0.82^aB^	25.98 ± 0.51^aA^
	60	12.88 ± 1.51^bA^	11.07 ± 0.51^cA^
	90	10.32 ± 0.65^bA^	9.27 ± 1.03^cA^
Total phenolics assay			
mg GAE/mL	1	0.17 ± 0.01^aA^	0.10 ± 0.00^aB^
	30	0.14 ± 0.01^aA^	0.06 ± 0.00^aB^
	60	0.20 ± 0.01^aA^	0.11 ± 0.00^aB^
	90	0.22 ± 0.01^aA^	0.12 ± 0.01^aB^

*Note*: The lower case letters in the same column (^a,b^) indicate that the differences between the ripening days of the same cheese sample are significant (*p* < .05). The upper case letters in the same line (^A,B^) indicate that the differences between B1 and B2 cheeses on the same ripening day are significant (*p* < .05).

Abbreviation: B1 and B2, Camis cheeses produced with two different traditional methods.

There was no statistically significant difference in TEAC values between B1 and B2 cheeses between ripening days and between B1 and B2 cheeses at all ripening days (*p* > .05). The difference in heat treatment of the milk and boiling the curd of the two cheeses did not cause a significant difference in ABTS values. However, considering the differences between the 1st and 90th ripening days, the increase in the activity of B1 cheese was more noticeable. Cooking the curd had positive effects on antioxidant activity, since Gouda, Ras, and Edam cheeses that were cooked after curd formation showed higher values of TEAC (Helal & Tagliazucchi, 2023), as in B1 cheese. In cheese ripening, the endogenous milk enzymes, the residual coagulants, and the enzymes of starter culture or non‐starter lactic acid bacteria (NSLAB) coming from raw milk, ingredients, or equipment play an essential role in the diversity of bioactive peptides (Santiago‐López et al., 2018). Heat treatment of the milk affects the specific sequence and amount of bioactive peptides formed in cheese by increasing the milk plasmin activity. Antioxidant peptides are formed resulting from secondary proteolysis by the action of plasmin and NSLAB enzymes during cheese ripening (Mushtaq et al., 2016), increasing the activity as in B1 cheese. The antioxidant activity of WSEs of white brined goat cheese (Barać et al., 2016) and Kalari cheese (Mushtaq et al., 2016) increased significantly during ripening. However, some of these peptides are also hydrolyzed as the ripening progresses, and these small peptides or amino acids may not possess antioxidant activity (Hossain et al., 2020), causing a decrease in later stages of ripening, as in B2 cheese. The increase in antioxidant activity during the ripening of Edirne Feta cheese produced from cow, sheep, and goat milk and the decrease after a certain ripening period were similar to B2 cheese (Halici Demir, 2021). The level of peptides formed varies depending on the equilibrium between the formation and further breakdown of them during ripening (Albenzio et al., 2017).

The % inhibition results of ABTS obtained from B1 and B2 cheeses (2.32–3.34%) were relatively close to that of Cheddar cheese from buffalo milk ripened for 90 days (1.76–7.92%, Huma et al., 2018), 30 days old Mozzarella cheese (1.53–5.15%, Saleem et al., 2023), nevertheless lower than 7 days old Kalari cheese (11.00%, Mushtaq et al., 2019), buffalo cheese ripened for 21 days (8.56–12.03%, Ribas et al., 2019), fresh Kradi cheese (12.03%, Mushtaq et al., 2021), fresh buffalo cheese (33.39%, Silva et al., 2019), buffalo Domiati cheese ripened for 90 days (29.14–38.93%, Taha et al., 2020), and 120 days old Cheddar cheese (28–85%, Hossain et al., 2020). An inconsistent tendency in % inhibition values is observed between ripening days in this study, as well as all these relevant studies, depending on the processing and ripening conditions whose effects were explained earlier.

When the DPPH results are examined (Table 5), it is observed that the DPPH radical scavenging activity of both cheese samples increased on day 30 and decreased in the later stages of ripening. There were statistically significant differences in DPPH inhibition rates (%) of B1 cheese between days 1–30, 30–60, and 30–90 of ripening, and B2 cheese between all the ripening days except 60 and 90th days (*p* < .05). In addition, the inhibition rates were statistically different between B1 and B2 cheese on days 1 and 30, but similar on days 60 and 90. DPPH inhibition rates of B2 cheese were higher than those of B1 cheese during ripening. It is seen that the second heat treatment applied, boiling the curd in B1 cheese production, had a negative effect on the DPPH radical scavenging activity of the Camis cheese. It may be related to the fact that heat treatment may reduce the antioxidant activity, probably due to the deterioration of natural antioxidant compounds in milk, like vitamins (Rinaldi et al., 2021). The DPPH inhibition rates obtained (8.54–26.34%) were close to the rates obtained for different cheeses such as 21‐day‐old buffalo cheese (4.86–13.08%, Ribas et al., 2019) and 150‐day‐old Kashar cheese (8.00–11.50%, Gurmeric et al., 2024), but higher than Mozzarella (2.52–5.15%, Saleem et al., 2023) and were affected by the ripening process as expressed before. An increase in antioxidant activity was observed until the 60th days of ripening in Cheddar (Hossain et al., 2020), Kashar cheese (Gurmeric et al., 2024), and 30th days in Mozzarella (Saleem et al., 2023), and then a decrease, which was thought to be due to the inability of antioxidant peptides to resist advanced proteolysis. In addition, activity changes were observed depending on the type of milk. The DPPH inhibition activity of cow Cheddar cheese (Hossain et al., 2020) was higher than that of Kalari cheese produced from raw buffalo milk (Mushtaq et al., 2016). On the other hand, Cheddar cheese produced from buffalo milk showed slightly higher inhibition rates than that produced from cow milk (Huma et al., 2018). The antioxidant activity of different types of cheese milk varies depending on milk fat (tocopherols, retinols, and carotenoids), caseins, whey proteins, thiol groups, ascorbate, and phenols (Abd El‐Fattah et al., 2020), thus the species‐based chemical composition. Khan et al. (2017) attributed the higher antioxidant activity of buffalo milk to the higher vitamin E and C, flavonoids, cysteine, tyrosine, selenium, and zinc contents compared to cow milk.

The antioxidant activity was also evaluated by a total phenolics assay, and the results were given in mg GAE mL^−1^ (Table 5). Considering the ripening time, the antioxidant activity of each B1 and B2 cheese was statistically similar (*p* > .05), while they statistically differed at each time‐measured point (*p* < .05). There was a slight decrease in 30th‐day samples, and the antioxidant activity increased until the end of ripening for both cheeses. Basil fortification increased the TPC of the fresh buffalo cheeses (Ribas et al., 2019), but a reduction was seen on day 21, as in the 30th ripening day in this study. It was observed that the curd boiling process has positive effects on the TPC of Camis cheese, as B1 cheese had higher TPC values (0.14–0.22 mg GAE m^−1^). The TPC of both B1 and B2 samples were lower than sheep cheese (0.21–0.25 mg GAE mL^−1^, Chávez‐Servín et al., 2018) and herby cheese (0.35–1.11 mg GAE g^−1^, Kara, 2019), but higher than Mozzarella (0.02–0.07 mg GAE mL^−1^, Yerlikaya et al., 2021). In Kashar cheese production, where there is a wet boiling method as in our B1 cheese, the TPC content of the samples was high (0.37 mg GAE mL^−1^, Kilic & Koyuncu, 2024). Nevertheless, it has been reported that high heat treatment may decrease the TPC of the products by the release, breakdown, and hydrolysis of the phenolics, and by causing complex reactions affecting the phenolic composition, and therefore the antioxidant activity (Chávez‐Servín et al., 2018). The authors stated that the TPC concentration of raw goat cheese was higher than that of pasteurized cheese. This inconsistency may occur due to milk type, composition, and the cheesemaking process, as expressed before.

The values obtained by the three methods used to determine antioxidant activity differed significantly. This difference may be due to the difference in the structure and reaction forms of the radicals used in the methods (Kara, 2019).

## 4 CONCLUSION

The antimicrobial and antioxidant properties of Camis cheeses, which were produced by two different traditional methods mainly differing in curd boiling, were compared. The WSEs of both cheeses had noticeable antimicrobial effects on *E. coli* (ATCC® 25922™) and *Staph. aureus* (ATCC® 23235™), but not very high antioxidant activity. The lack of starter culture, which contributes to the ripening process by proteolysis and releases the antimicrobial and antioxidative peptides, explains the low values determined. B1 cheese showed higher antimicrobial and antioxidant activity during ripening, indicating that boiling the curd has a favorable effect on bioactivity. It would be appropriate to say that the consumption of extracts of traditional Camis cheese or purified bioactive peptides or their use in functional food products is important in terms of eliminating the negative effects encountered with the use of synthetic antimicrobial and antioxidant components to provide the desired health effect from natural sources. However, further studies are needed for the isolation of bioactive peptides from traditional Camis cheeses and their characterization by chromatographic methods.

## CONFLICT OF INTEREST STATEMENT

There is no conflict of interest to declare.

## Data Availability

The data that support the findings of this study are available from the corresponding author upon reasonable request.
